# Mitochondrial superoxide dismutase 2 mediates γ-irradiation-induced cancer cell invasion

**DOI:** 10.1038/s12276-019-0207-5

**Published:** 2019-02-12

**Authors:** Chan-Hun Jung, Eun Mi Kim, Jie-Young Song, Jong Kuk Park, Hong-Duck Um

**Affiliations:** 1 Division of Radiation Biomedical Research, Korea Institute of Radiological and Medical Sciences, Seoul, 01812 Korea; 2 Present Address: Jaseng Spine and Joint Research Institute, Jaseng Medical Foundation, Seoul, Korea

**Keywords:** Oncogenes, Cell invasion

## Abstract

Sublethal doses of γ-rays promote cancer cell invasion by stimulating a signaling pathway that sequentially involves p53, sulfatase 2 (SULF2), β-catenin, interleukin-6 (IL-6), signal transducer and activator of transcription 3 (STAT3), and Bcl-X_L_. Given that Bcl-X_L_ can increase O_2_^•−^ production by stimulating respiratory complex I, the possible role of mitochondrial reactive oxygen species (ROS) in γ-irradiation-induced cell invasion was investigated. Indeed, γ-irradiation promoted cell invasion by increasing mitochondrial ROS levels, which was prevented by metformin, an inhibitor of complex I. γ-Irradiation-stimulated STAT3 increased the expression of superoxide dismutase 2 (SOD2), a mitochondrial enzyme that catalyzes the conversion of O_2_^•−^ to hydrogen peroxide (H_2_O_2_). In contrast to O_2_^•−^, H_2_O_2_ functions as a signaling molecule. γ-Irradiation consistently stimulated the Src-dependent invasion pathway in a manner dependent on both complex I and SOD2. SOD2 was also essential for the invasion of un-irradiated cancer cells induced by upregulation of Bcl-X_L_, an intracellular oncogene, or extracellular factors, such as SULF2 and IL-6. Overall, these data suggested that SOD2 is critical for the malignant effects of radiotherapy and tumor progression through diverse endogenous factors.

## Cancer treatment: Stopping the spread after radiotherapy

A drug usually used to treat type 2 diabetes may also help to prevent cancer relapse following radiotherapy, which is commonly used to kill cancer cells. However, any tumor cells that survive radiation are highly invasive, sometimes causing tumors to spread. Hong-Duck Um and co-workers at the Korea Institute of Radiological & Medical Sciences in Seoul, South Korea, noticed that the surviving cells often showed higher levels of a key enzyme, superoxide dismutase 2 (SOD2), which is involved in energy production in the cellular powerhouse, the mitochondria. Artificially increasing levels of SOD2, without radiation, made cells more invasive. Treatment with metformin, which prevents production of the molecule that SOD2 acts on, prevented cells from becoming invasive. SOD2 has been implicated in many cancers, and is therefore a very promising therapeutic target.

## Introduction

Ionizing radiation (IR), such as γ-irradiation, is a major therapeutic modality for treating cancer. In most patients, IR offers a significant survival benefit, but in some patients, local recurrence or distal metastasis following radiotherapy is a major therapeutic challenge. These undesirable consequences may reflect the regrowth or spread of cancer cells that survived radiotherapy. Studies using cultured cells and animal models have shown that sublethal doses of IR increase the mobility, invasiveness, and metastatic potential of cancer cells^1,2^, suggesting that IR promotes malignant behavior in postradiation tumors. Therefore, the cellular components involved in the malignant effects of IR should be defined to develop new strategies for improving the therapeutic effects of IR.

Mitochondria have emerged as central regulators of cancer cell invasion and metastasis, and reactive oxygen species (ROS) produced via the mitochondrial respiratory chain have been implicated as stimulators of various cellular pathways leading to cell migration and invasion^3^. The production of mitochondrial ROS is regulated by Bcl-2 family proteins^4^. Although they were originally identified as key regulators of cell death^5^, certain Bcl-2 family members also regulate cell migration, invasion, and cancer metastasis^4^. A well-characterized example is the group of pro-survival Bcl-2 family members, including Bcl-X_L_, Bcl-2, and Bcl-w, which stimulate complex I, a major source of ROS in the mitochondrial respiratory chain, to produce additional ROS. The ROS produced the following overexpression of Bcl-w, or Bcl-X_L_ promote cell invasion by stimulating Src and its downstream signaling components^6^.

We have previously shown that sublethal doses of IR increase sulfatase 2 (SULF2) expression via the p53 transcription factor^7^. SULF2 is an extracellular sulfatase that modulates the signaling activities of diverse cell surface receptors^8^, and IR-induced SULF2 mediates the pro-invasive activity of IR by stimulating the signaling pathway that sequentially involves β-catenin, interleukin-6 (IL-6), and signal transducer and activator of transcription 3 (STAT3)^7^. STAT3 is a transcription factor that induces Bcl-X_L_ expression^9^. Consistently, sublethal doses of IR increase the messenger RNA (mRNA) and protein levels of Bcl-X_L_ in several cancer cell types, and Bcl-X_L_ knockdown abolishes the pro-invasive activity of IR^7,10^, suggesting a role for Bcl-X_L_ in IR-induced cell invasion. These results suggest the involvement of mitochondrial ROS in IR-induced cancer cell invasion. However, this possibility has not been directly addressed.

ROS include free radicals, such as superoxide anion (O_2_^•−^) and hydroxyl radical (HO^•^), as well as nonradical molecules, such as hydrogen peroxide (H_2_O_2_). Among these free radicals, H_2_O_2_ has a relatively long half-life and can freely diffuse to induce signaling^11^. Therefore, it is thought that H_2_O_2_ is the effector ROS that modulates activities of signaling molecules. However, the mitochondrial respiratory chain produces O_2_^•−^ that needs to be converted to H_2_O_2_ to modulate signaling. Superoxide dismutase (SOD) is a metalloenzyme that catalyzes the conversion of O_2_^•−^ to H_2_O_2_. In mammals, there are three distinct types of SOD as follows: Cu/ZnSOD (SOD1), MnSOD (SOD2), and extracellular Cu/ZnSOD (SOD3)^12^. SOD1 is the major intracellular form of SOD, and it is localized primarily in the cytosol. In contrast, SOD2 is exclusively localized to the mitochondrial matrix. This feature of SOD2 suggests that it may be involved in the conversion of mitochondrial O_2_^•−^ to H_2_O_2_, thus contributing to cell invasion. Hence, the present study investigated the potential role of SOD2 in IR-induced cell invasion. The possibility was indeed supported by our data. The present findings demonstrated that SOD2 also mediates the invasion of un-irradiated cancer cells induced by upregulation of diverse oncogenic components, supporting the role of SOD2 in tumor progression. Therefore, SOD2 is a potential target for preventing cancer cell invasion following radiotherapy and suppressing tumor progression under diverse conditions.

## Materials and methods

### Antibodies and recombinant proteins

The following antibodies were used in the present study: anti-SOD2 from Enzo Life Sciences (Farmingdale, NY, USA); anti-IL-6 and anti-β-catenin from Santa Cruz Biotechnology (Santa Cruz, CA, USA); anti-Bcl-X_L_, anti-STAT3, anti-Src, and anti-phospho-Src from Cell Signaling Technology (Danvers, MA, USA); and anti-β-actin from Sigma-Aldrich (St. Louis, MO, USA). Recombinant human IL-6 was purchased from Millipore (Darmstadt, Germany).

### siRNA and shRNAs

Small interfering RNA (siRNAs) targeting IL-6 (S7312) and Bcl-X_L_ (120717) were purchased from Ambion (Austin, TX, USA). siRNAs targeting SOD2 (sc-41655), β-catenin (sc-44275), and STAT3 (sc-29209) as well as lentiviruses expressing small hairpin RNAs (shRNAs) targeting SOD2 (sc-41655-V), Bcl-X_L_ (sc-43630-V), and SULF2 (sc-63088-V) were obtained from Santa Cruz Biotechnology.

### Cell culture, transfection, infection, and treatments

All cell lines used in this study, except for HCT116/p53^wt^ and HCT116/p53(−/−) colon cancer cells (a generous gift from Dr. Bert Vogelstein), were obtained from the American Type Culture Collection (Manassas, VA, USA). Cells were cultured in RPMI-1640 (A549, H1299, and H460 lung cancer cells) or DMED (MCF-7 breast cancer and HCT116 cells) medium supplemented with 10% heat-inactivated fetal bovine serum (FBS). Expression constructs for SOD2, SULF2, and Bcl-X_L_ were prepared using the pcDNA3 vector (Invitrogen, Carlsbad, CA, USA). Expression constructs and siRNAs were introduced into cells using Lipofectamine 2000 (Invitrogen) according to the manufacturer’s protocol. The transfectants were used for experiments after 48 h of recovery. For long-term gene silencing, cells were infected with lentiviruses containing shRNAs targeting SOD2 in the presence of polybrene (5 μg/mL) according to the manufacturer’s protocols. Infected cells were selected with puromycin (2 μg/mL). For irradiation, cells were exposed to the specified doses of γ-rays from a ^137^Cs γ-ray source (Atomic Energy of Canada, Chalk River, Canada) at a dose rate of 3 Gy/min. Alternatively, cells were treated with the indicated concentrations of IL-6.

### Western blotting

Cell lysates were prepared using previously described methods^13^. Proteins in the lysates were separated by sodium dodecyl sulfate-polyacrylamide gel electrophoresis, electrotransferred onto nitrocellulose membranes (Millipore), and analyzed using the specified antibodies and an ECL detection system (Bio-Rad, Hercules, CA, USA).

### RT-PCR and quantitative real-time PCR

Reverse transcription-PCR (RT-PCR) was performed by amplifying complementary DNA (cDNA) in Premix PCR solution (Takara, Shiga, Japan) with SOD2 primers (5′-GGA-AGC-CAT-CAA-ACG-TGA-CTT-3′ and 5′-GTG-CTC-CCA-CAC-ATC-AAT-CC-3′). Quantitative real-time PCR was performed using the SYBR Fast Universal qPCR Kit (Kapa Biosystems, Wilmington, MA, USA) and SOD2 primers (5′-GGC-CTA-CGT-GAA-CAA-CCT-GAA-3′ and 5′-CTG-TAA-CAT-CTC-CCT-TGG-CCA-3′). *GAPDH* was amplified in both PCR assays with the following primers as an internal control for normalization: 5′-CAT-CTC-TGC-CCC-CTC-TGC-TGA-3′ and 5′-GGA-TGA-CCT-TGC-CCA-CAG-CCT-3′. The RT-PCR and real-time PCR results were analyzed by agarose gel electrophoresis and an IQ-5 Real-Time System (Bio-Rad), respectively.

### Invasion assay

As described previously^14^, cells in serum-free medium were seeded onto the upper surfaces of Matrigel-coated Transwell chambers (BD Biosciences, San Jose, CA, USA). The lower compartments of the chambers were filled with medium supplemented with 10% heat-inactivated FBS. After 16 h of incubation, cells that invaded the lower surface of the filter were stained with the Diff-Quick Kit (Fisher Scientific, Waltham, MA, USA) and counted under a microscope.

### Analysis of mitochondrial ROS levels

Cells were exposed to 10 μM MitoSOX Red (Invitrogen) or 5 μM Peroxy Orange-1 (Tocris Bioscience, Bristol, UK) for 30 min, and cell-associated fluorescence was analyzed by flow cytometry.

### Clonogenic assay

Various numbers of cells infected with the specified lentiviruses were seeded in triplicate into 60 mm dishes (100, 200, 400, and 800 cells/dish). After 24 h of incubation, cells were exposed to different doses of γ-rays (1, 3, 5, and 7 Gy). Irradiated and untreated control cells were cultured for 14 days. The number of colonies was counted with a colony counter (Imaging Products, Hollywood, CA, USA), and clonogenic survival was calculated as described previously^15^.

### Statistical analysis

All experiments were performed at least three times to obtain means and standard deviations. Statistical significance was determined with one-way analysis of variance (GraphPad Software, La Jolla, CA, USA), and *p* values <0.05 were considered significant.

## Results

### Sublethal doses of IR increase SOD2 expression via the p53/SULF2/β-catenin/IL-6/STAT3 pathway

**Fig. 1:**
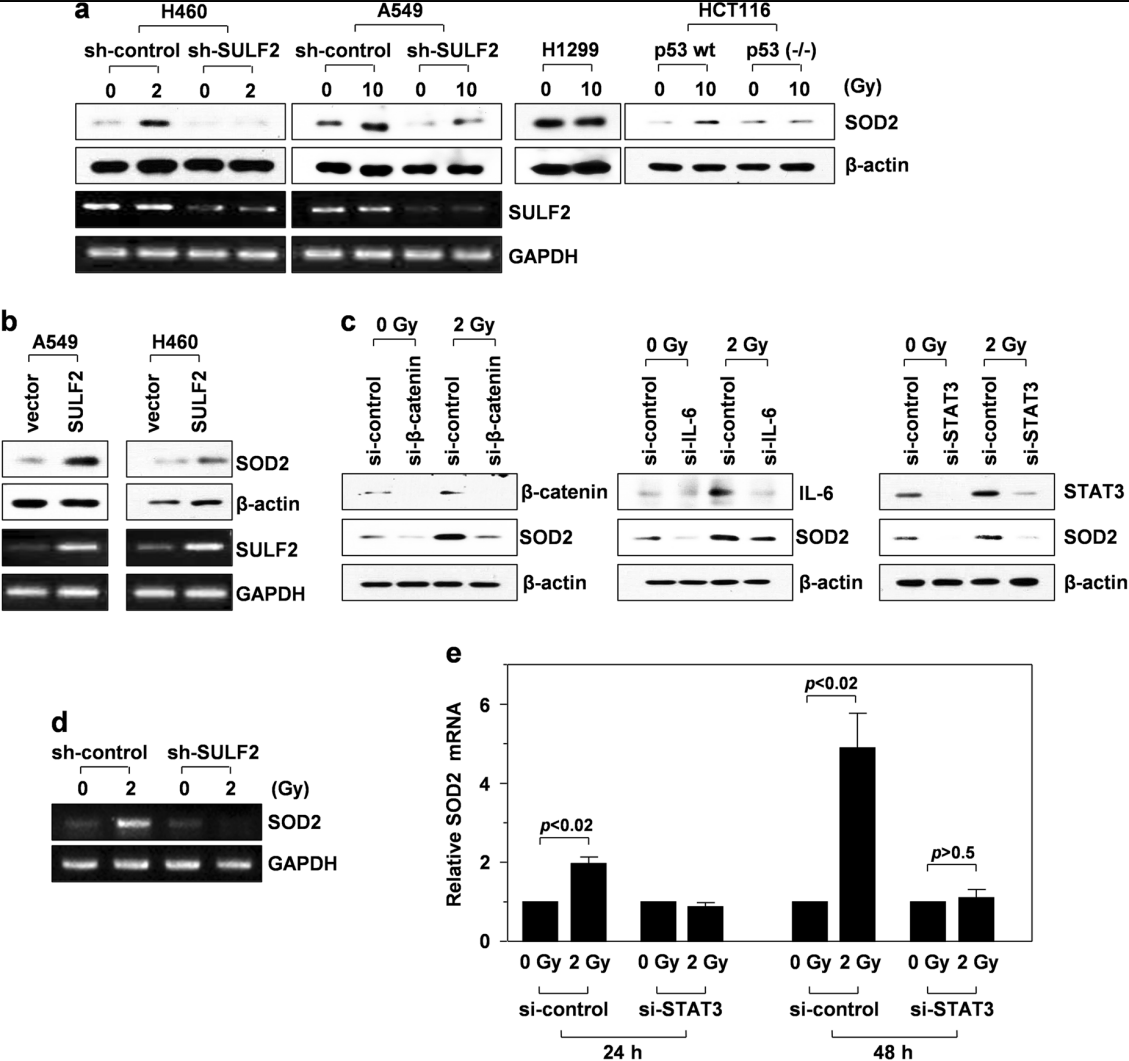
IR induces SOD2 expression via the p53/SULF2/β-catenin/IL-6/STAT3 pathway. **a–d** Western blotting and RT-PCR were performed 48 h after γ-irradiation. **a** H460 and A549 lung cancer cells (p53^wt^) were infected with lentiviruses expressing control (nontargeting sequence) or SULF2-specific shRNA. These transfectants, along with H1299 lung cancer cells (p53^null^) and p53^wt^-expressing or p53-knockout HCT116 colon cancer cells, were irradiated with the indicated doses of γ-rays, and SOD2 levels were compared by western blot analysis using β-actin as a loading control. SULF2 expression was compared by RT-PCR using GAPDH as a loading control. **b** A549 and H460 cells were transfected with an empty or SULF2 expression vector, and SOD2 protein and SULF2 mRNA levels were compared. **c** H460 cells treated with a control or an siRNA targeting β-catenin, IL-6, or STAT3 were irradiated with 2 Gy of γ-rays, and the levels of the indicated proteins were compared. **d** H460 cells infected with the lentiviruses indicated in **a** were irradiated, and SOD2 mRNA levels were analyzed by RT-PCR. **e** H460 cells treated with a control or a STAT3-targeting siRNA were irradiated, and SOD2 mRNA levels were compared by quantitative real-time PCR at 24 and 48 h after irradiation

To investigate the potential involvement of SOD2 in IR-induced cell invasion, p53^wt^-expressing (H460 and A549 lung cancer cells as well as HCT116 colon cancer cells) and p53^null^ cells (H1299 lung cancer cells) were irradiated with sublethal doses of γ-rays. Irradiation elevated protein levels of SOD2 in the p53^wt^-expressing cells but not in the p53^null^ cells (Fig. 1a). Consistently, knockout of p53 in HCT116 cells abolished IR-induced SOD2 accumulation. It has been previously confirmed that p53 protein levels in p53^wt^-expressing cells are elevated upon γ-irradiation, but that p53 expression is not detected in p53^null^ or p53-knockout cells even after γ-irradiation^16–18^. These findings suggested that the γ-irradiation mediated increase in SOD2 levels is p53 dependent.

p53 mediates IR-induced cell invasion by stimulating cellular pathways sequentially involving SULF2, β-catenin, IL-6, and STAT3^7^. To investigate the relationship between this pathway and SOD2 induction, SULF2 was knocked down in H460 and A549 cells using a specific shRNA, which abolished or attenuated IR-induced SOD2 accumulation (Fig. 1a). Consistently, SOD2 protein levels were increased following overexpression of SULF2 in un-irradiated cells (Fig. 1b), confirming that SULF2 increases SOD2 protein levels. Moreover, knockdown of β-catenin, IL-6, or STAT3 using specific siRNAs reduced IR-induced accumulation of SOD2 protein (Fig. 1c), suggesting that IR increases SOD2 levels via the p53/SULF2/β-catenin/IL-6/STAT3 pathway.

In addition, IR elevated mRNA levels of SOD2, and this increase was abolished by knockdown of SULF2 (Fig. 1d) or STAT3 (Fig. 1e), suggesting that IR induces SOD2 protein accumulation by increasing its mRNA levels via STAT3 stimulated by the SULF2 pathway. The ability of STAT3 to induce SOD2 gene expression has also been reported in a mouse model of ischemic brain injury^19^. Moreover, the promoter region of SOD2 has been shown to contain STAT3-binding sites^19^.

### SOD2 mediates IR-induced cell invasion

**Fig. 2:**
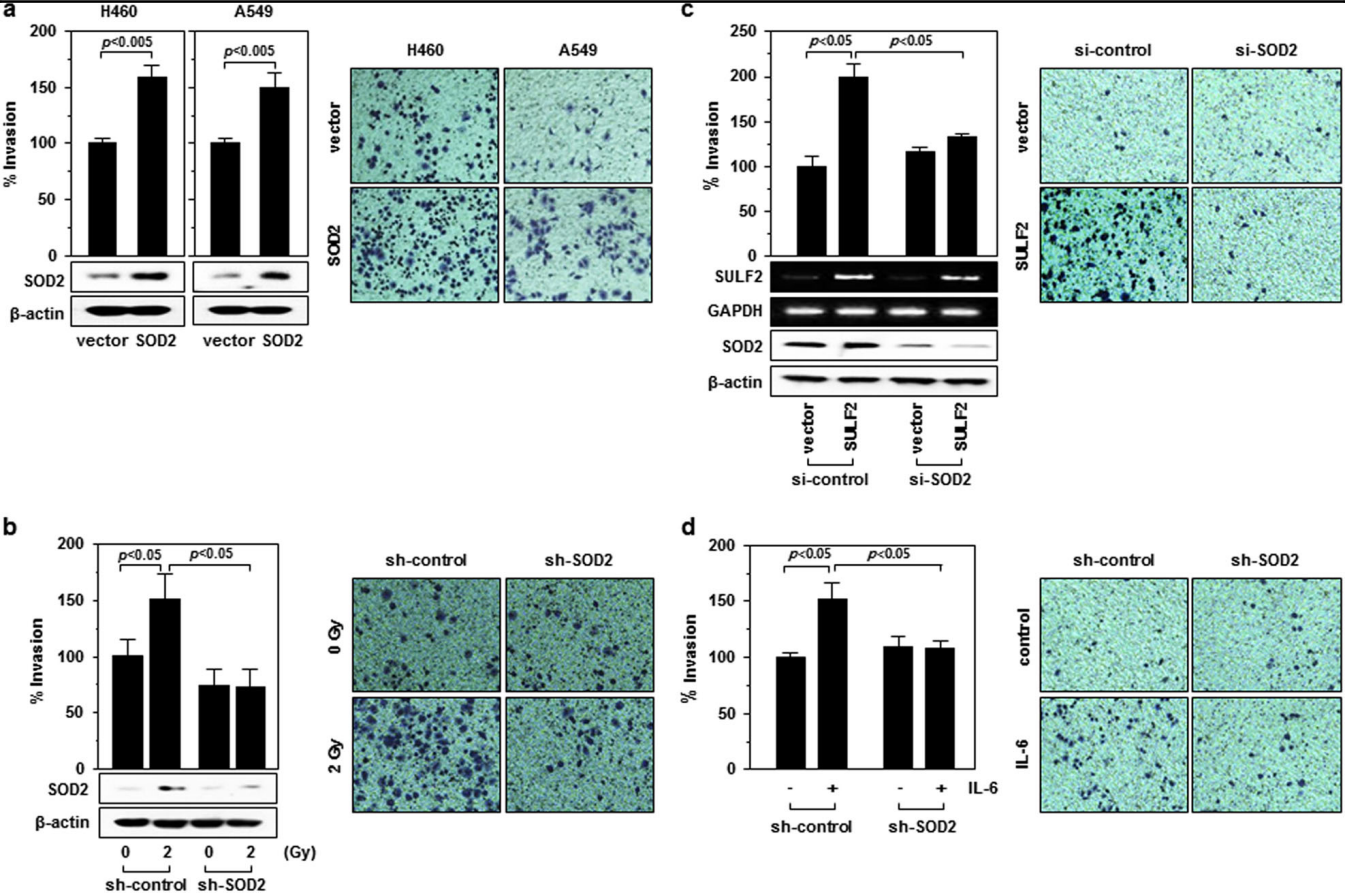
SOD2 mediates IR-induced cell invasion. **a** H460 and A549 cells were transfected with control (empty vector) or SOD2 expression vector. After 48 h of incubation, a cell invasion assay was performed. Cells that invaded through Matrigel-coated polycarbonate filters were imaged, and the number of invaded cells was counted and plotted. SOD protein levels were compared by western blot analysis. **b** H460 cells infected with lentiviruses expressing a control or SOD2-targeting shRNA were irradiated, and invasiveness and protein levels were then analyzed 48 h after irradiation. **c** H460 cells were cotransfected with a SULF2 expression vector and SOD2 siRNA as per the indicated combinations, and cell invasiveness was then assessed. **d** H460 cells infected with lentiviruses expressing control RNA or SOD2-specific shRNA were incubated in the presence or absence of IL-6 (100 ng/mL) for 24 h. Cell invasiveness was then assessed

To investigate the potential role of SOD2 in IR-induced cell invasion, SOD2 was overexpressed in H460 and A549 cells. The invasiveness of both cell lines was enhanced by SOD2 overexpression (Fig. 2a). Consistently, IR-induced cell invasion was reduced by knockdown of SOD2 (Fig. 2b), suggesting that SOD2 mediates IR-induced cell invasion. Consistent with the role of SOD2, its knockdown also abolished cell invasion induced by SULF2 overexpression (Fig. 2c) or IL-6 treatment (Fig. 2d).

### IR induces activation of the mitochondrial ROS/Src-dependent invasion pathway

**Fig. 3:**
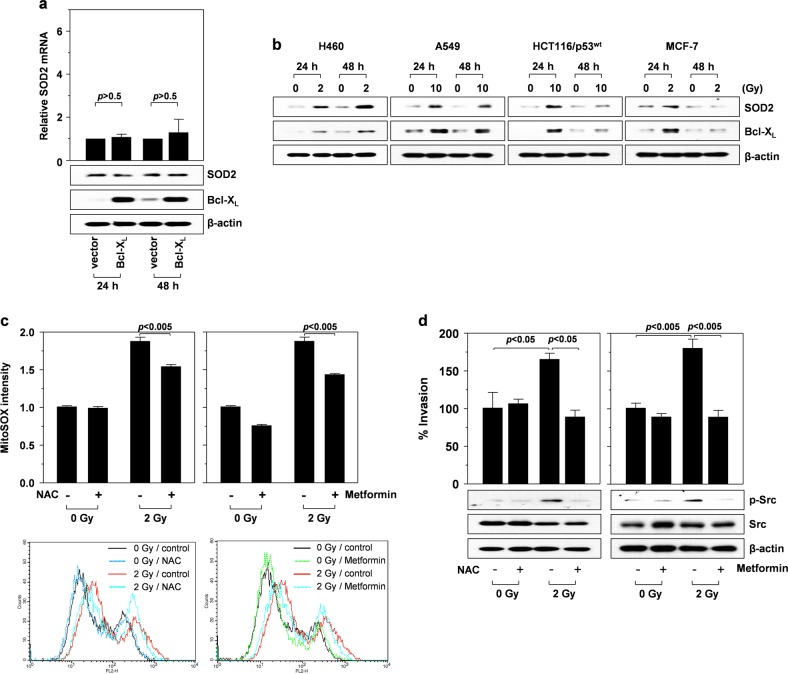
IR induces the mitochondrial ROS/Src-dependent invasion pathway. **a** H460 cells were transfected with a control or a Bcl-X_L_-expression vector. After 24 and 48 h of incubation, SOD2 mRNA levels were compared by quantitative real-time PCR (top), and the levels of the indicated proteins were compared by western blotting (bottom). **b** The indicated cells were irradiated with the specified doses of γ-rays. Western blotting for SOD2 and Bcl-X_L_ was performed at 24 and 48 h after irradiation. **c** Irradiated (2 Gy, 48 h) and untreated control H460 cells were incubated in the presence or absence of NAC (5 mM) and metformin (5 mM) for 1 h, and mitochondrial ROS levels were compared by staining cells with MitoSOX Red probes and analyzing them by flow cytometry. **d** Irradiated and untreated control cells were analyzed for their invasiveness in the presence or absence of NAC or metformin. The levels of Src and phosphorylated Src in the cells were compared by western blotting

In response to IR, STAT3 increases the mRNA and protein levels of Bcl-X_L_, which is essential for IR-induced cell invasion^10^. To eliminate the possibility of Bcl-X_L_ influencing SOD2 expression, Bcl-X_L_ was overexpressed in H460 cells. Bcl-X_L_ overexpression did not significantly affect SOD2 mRNA and protein levels (Fig. 3a), confirming the irrelevance of Bcl-X_L_ in SOD2 induction. These data suggested that STAT3 promotes the expression of Bcl-X_L_ and SOD2 in response to γ-irradiation. Notably, these two proteins co-accumulated at 24 and/or 48 h postradiation (Fig. 3b). This phenomenon was observed in four different p53^wt^ cancer cell lines, including lung (H460 and A549), colon (HCT116/p53^wt^), and breast cancer cells (MCF-7), and the post-irradiation peak accumulation time was cell type-dependent. The co-induction of Bcl-X_L_ and SOD2 implied their cooperation in IR-induced cellular responses.

**Fig. 4:**
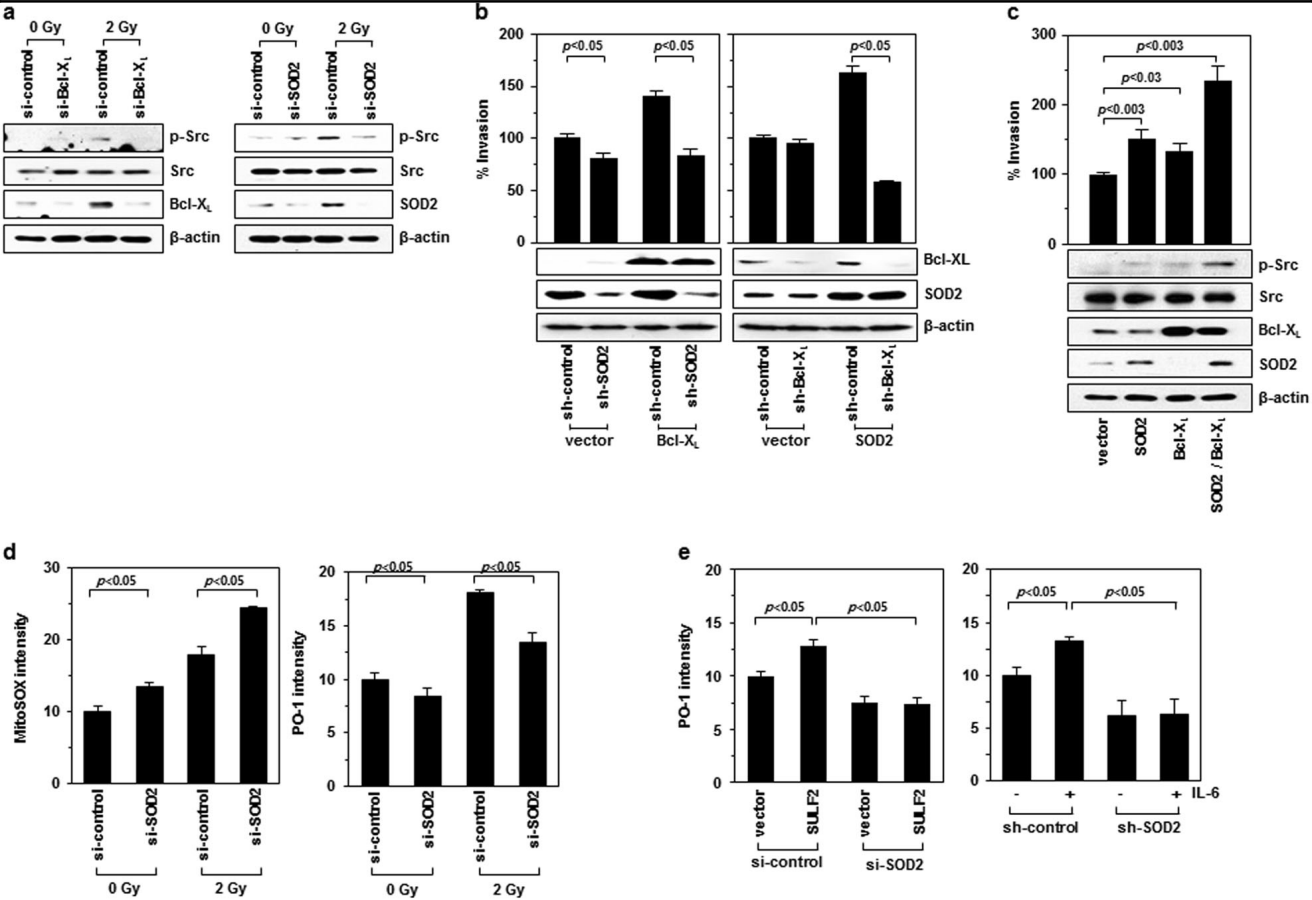
Bcl-XL and SOD2 functionally cooperate to promote Src phosphorylation and cell invasion. **a** H460 cells treated with a control, Bcl-X_L_-targeting, or SOD2-targeting siRNA were irradiated, and the levels of the indicated proteins were compared 24 h after irradiation. **b** H460 cells infected with lentiviruses expressing the specified shRNAs were transfected with a Bcl-X_L_ or SOD2 expression vector in the indicated combinations. Cellular invasiveness and the levels of Bcl-X_L_ and SOD2 were compared after 48 h of incubation. **c** H460 cells were transfected with a Bcl-X_L_ or SOD2 expression vector in the indicated combinations. Cellular invasiveness and the levels of the indicated proteins were compared after 48 h of incubation. **d** H460 cells treated with a control or SOD2-targeting siRNA were irradiated (2 Gy). After 48 h of incubation, irradiated and untreated control cells were analyzed for their levels of O_2_^•−^ and H_2_O_2_ using MitoSox Red and Peroxy Orange-1, respectively. **e** H460 cells were transfected with a SULF2 expression vector and SOD2-targeting siRNA in the indicated combinations (left). Alternatively, H460 cells infected with lentiviruses expressing a control or SOD2-targeting shRNA were incubated in the presence or absence of 100 ng/mL IL-6 (right). After treatment for 24 h, cellular H_2_O_2_ levels were compared using Peroxy Orange-1

Bcl-X_L_ overexpression increases the ability of complex I to produce ROS, which, in turn, stimulates the Src-dependent invasion pathway^6^. Given that IR upregulates Bcl-X_L_^10^, it may also stimulate the ROS/Src pathway. Because complex I produces O_2_^•−^, the levels of mitochondrial O_2_^•−^ levels in control and irradiated cells were compared using MitoSox Red, a probe specific for mitochondrial O_2_^•−^. IR increased mitochondrial O_2_^•−^ levels, which was prevented by *N*-acetylcysteine (NAC), an ROS scavenger, or metformin, an inhibitor of complex I^20^ (Fig. 3c), suggesting that complex I contributes to IR-induced O_2_^•−^ production. IR also increased Src phosphorylation, and both IR-induced cell invasion and Src phosphorylation were abolished by NAC or metformin (Fig. 3d). Similar effects on IR-induced Src phosphorylation were observed following Bcl-X_L_ knockdown (Fig. 4a, left). These results suggested that O_2_^•−^ produced by the Bcl-X_L_/complex I pathway contributes to the ability of IR to promote the Src-dependent invasion pathway.

### SOD2 mediates IR-induced Src phosphorylation in a cooperative manner with Bcl-X_L_

While complex I produces O_2_^•−^, H_2_O_2_ is the ROS that stimulates Src phosphorylation^21^. Therefore, SOD2 may mediate IR-induced cell invasion by converting O_2_^•−^ to H_2_O_2_. IR-induced Src phosphorylation was attenuated by SOD2 knockdown (Fig. 4a, right). SOD2 knockdown also abolished Bcl-X_L_-induced cell invasion (Fig. 4b, left), a result consistent with the view that SOD2 acts downstream of Bcl-X_L_ to promote cell invasion. However, cell invasion induced by SOD2 overexpression was also prevented by Bcl-X_L_ knockdown (Fig. 4b, right), suggesting that SOD2 requires Bcl-X_L_ for cell invasion. The functional interdependence of Bcl-X_L_ and SOD2 supports the view that both O_2_^•−^ production (by Bcl-X_L_) and its conversion to H_2_O_2_ (by SOD2) are critical for cell invasion. The functional cooperation between Bcl-X_L_ and SOD2 was further supported by the finding that overexpression of both Bcl-X_L_ and SOD2 was superior to overexpression of either alone for inducing Src phosphorylation and cell invasion (Fig. 4c).

To directly confirm the role of SOD2 in the conversion of IR-induced O_2_^•−^ to H_2_O_2_, the levels of O_2_^•−^ and H_2_O_2_ were determined using MitoSox Red and Peroxy Orange-1^22^, respectively. SOD2 knockdown increased the amount of O_2_^•−^ and decreased H_2_O_2_ levels in both control and irradiated cells (Fig. 4d). Consistently, SULF2 overexpression and IL-6 treatment increased H_2_O_2_ levels, and this increase was prevented by SOD2 knockdown (Fig. 4e). Therefore, these findings clearly demonstrated that SOD2 contributes to the conversion of O_2_^•−^ generated via the IR-induced SULF2/IL-6 pathway to H_2_O_2_.

### SOD2 promotes cell invasion without altering radiosensitivity

**Fig. 5:**
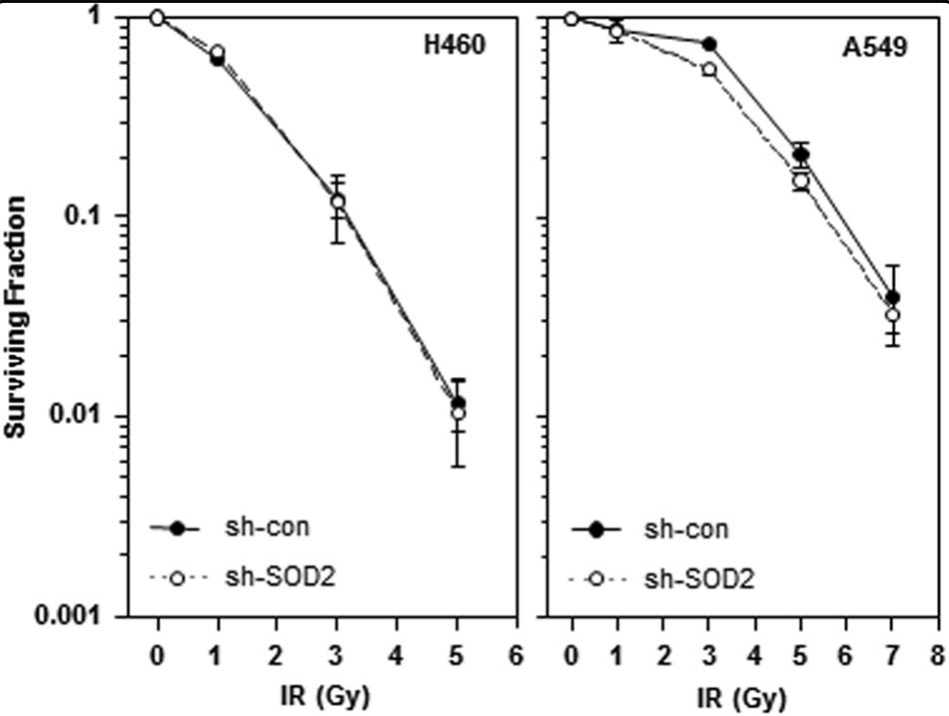
SOD2 does not influence radiosensitivity of H460 and A549 cells. H460 and A549 cells were infected with lentiviruses expressing a control or SOD2-targeting shRNA and irradiated with the indicated doses of γ-rays, and cell survival was then analyzed by clonogenic assays

SOD2 contributes to radioresistance in certain but not all cell types^23,24^. Colony-forming assays revealed that SOD2 knockdown did not significantly influence the radiosensitivity of H460 and A549 cells (Fig. 5). However, IR-induced cell invasion was inhibited by SOD2 knockdown (Fig. 2b), suggesting that SOD2 mediates IR-induced cell invasion without altering cellular radiosensitivity.

## Discussion

In the present study, overexpression or knockdown SOD2 in lung cancer cells showed that SOD2 promotes cancer cell invasion. As similar results have been reported for other types of cancer, such as fibrosarcoma^25^, salivary^26^, and tongue carcinomas^27,28^, the pro-invasive activity of SOD2 appears to be a common feature of diverse cancers. This view is consistent with the report that SOD2 is upregulated in many cancer types, including lung, breast, colon, brain, thyroid, gastric, and salivary cancers, and especially late-stage metastatic cancers^12^. SOD2 upregulation in cancer cells has been correlated with distant metastasis, poorer prognosis, and lower overall and disease-free survival^12,28^. Therefore, SOD2 upregulation is critical for tumor progression.

**Fig. 6:**
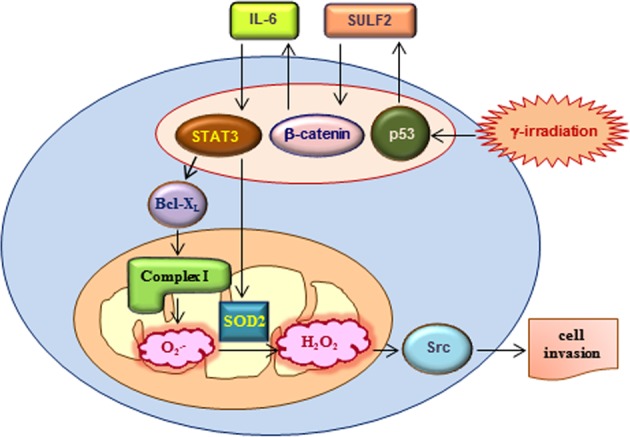
Schematic model for the role of mitochondria in IR-induced cell invasion. STAT3 stimulated by IR via the p53/SULF2/β-catenin/IL-6 pathway induces Bcl-X_L_ and SOD2 expression. Bcl-X_L_ increases the ability of complex I to produce O_2_^•−^, and SOD2 converts mitochondrial O_2_^•−^ to H_2_O_2_, which, in turn, acts as a signaling molecule to promote cell invasion

An important finding of the present study was that SOD2 functions as a key mediator of IR-induced cancer cell invasion, which was initially indicated by the finding that SOD2 mRNA and protein levels were elevated in cancer cells that survived γ-irradiation. In addition, the ability of IR to increase SOD2 expression has been reported in human fibroblasts^29^ as well as in mouse brain and gut^30^. In the present study, the IR-induced SOD2 expression was mediated by the p53/SULF2/β-catenin/IL-6/STAT3 pathway, which has been previously shown to mediate IR-induced cancer cell invasion^7^. Moreover, prevention of IR-induced cancer cell invasion by SOD2 knockdown directly supports the role of SOD2 in the malignant effects of sublethal doses of IR. This finding and our previous findings^7,10^ demonstrate that IR-stimulated STAT3 promotes cancer cell invasion by inducing the expression of SOD2 and Bcl-X_L_. Based on the recent report that Bcl-X_L_ upregulation increases the ability of complex I to produce ROS, which then stimulate the Src-dependent invasion pathway^6^, the ability of IR to utilize the mitochondrial pathway and promote cancer cell invasion was investigated. The findings demonstrated that IR increased mitochondrial ROS production and Src phosphorylation. Notably, ROS production, Src phosphorylation, and IR-induced cell invasion were prevented by treatment with an ROS scavenger (NAC) or a complex I inhibitor (metformin), thereby supporting the view that IR stimulates the Src-dependent invasion pathway by promoting complex I-dependent ROS production. Thus, SOD2 likely supports IR-induced cell invasion by converting O_2_^•−^ generated by complex I to H_2_O_2_, a signaling molecule that stimulates Src phosphorylation^21^, which was supported by the finding that SOD2 knockdown abolished IR-induced Src phosphorylation. Similar effects were observed when Bcl-X_L_ was knocked down, indicating that both Bcl-X_L_ and SOD2 are required for IR-mediated induction of the Src-dependent invasion pathway. Thus, IR-induced Bcl-X_L_ may increase the ability of complex I to produce O_2_^•−^, which is then converted to H_2_O_2_ by SOD2 (Fig. 6). The ability of SOD2 to convert IR-induced O_2_^•−^ to H_2_O_2_ was further confirmed by determining levels of O_2_^•−^ and H_2_O_2_ using specific dyes, namely, MitoSox Red and Peroxy Orange-1, respectively. According to the present model, SOD2 acts downstream of Bcl-X_L_ in an IR-induced signaling pathway, leading to cell invasion. However, despite this hierarchy, the functional relationship of Bcl-X_L_ and SOD2 was cooperative. Both Bcl-X_L_ and SOD2 were required to promote cell invasion, and their co-expression induced Src phosphorylation and cell invasion much more effectively than either alone, suggesting that both O_2_^•−^ production and its conversion to H_2_O_2_ are required for the pro-invasive activity of IR. The present data also suggested that IR fulfils these two requirements by co-inducing Bcl-X_L_ and SOD2 via STAT3. This co-induction was verified in lung, colon, and breast cancer cells. Therefore, the model depicted in Fig. 6 is a general mechanism applicable to diverse cancer types.

The ability of p53 to induce SOD2 expression has been reported by other investigators^31,32^. Although the consensus p53-binding sequences have been identified in the promoter region of SOD2^31^, they are not required for the transcriptional activation of SOD2 by p53^32^, suggesting that p53 induces SOD2 expression indirectly, which is consistent with the present model (Fig. 6).

Some investigators have reported that SOD2 protects cells from IR^23^, while others have reported that SOD2 is irrelevant in radioresistance^24^. While the reason for this discrepancy is still unclear, the present study demonstrated that SOD2 did not significantly influence the radiosensitivity of H460 and A549 cells. Considering that SOD2 plays an essential role in the IR-induced invasion of H460 cells, it is clear that SOD2 mediates IR-induced cell invasion without altering cellular radiosensitivity.

The present data also suggested that the pro-invasive role of SOD2 is not restricted to extrinsic treatments, such as radiotherapy, as it was also involved in intrinsic factor-induced cell invasion. In the present study, SOD2 was essential for cell invasion induced by Bcl-X_L_, an oncogene upregulated in many cancers^4^. In addition, SOD2 mediated cell invasion induced by extracellular factors, such as IL-6 and SULF2, suggesting that SOD2 plays an essential role in cell invasion induced in response to tumor microenvironment changes. Thus, SOD2 is critical for tumor progression under diverse conditions.

Metformin is a first-line medication for patients with type 2 diabetes because it reduces hyperglycemia by suppressing hepatic gluconeogenesis^33^. However, accumulating evidence has supported the potential of metformin for cancer therapy. Metformin has been shown to inhibit the growth of many cancer types^34^, and among cancer patients with diabetes, metformin users show better survival than non-users^35^. Metformin also improves tumor responses to radiotherapy by acting as a radiosensitizer^36^. As metformin is an inhibitor of complex I^20^, the ability of this drug to disrupt the malignant actions of IR was investigated. This possibility was supported by the finding that metformin suppressed the ability of IR to increase mitochondrial ROS production, Src phosphorylation, and cellular invasiveness. The present findings supported the view that metformin may improve the therapeutic effects of IR not only by acting as a radiosensitizer but also by preventing malignant actions of IR.

In conclusion, the present study showed that SOD2 is a key mediator of IR-induced cancer cell invasion, thereby supporting the critical role of mitochondria in cancer cell invasion and metastasis^4^. Considering the short half-life of O_2_^•− 11^, mitochondrial SOD2 may have an advantage for converting mitochondrial O_2_^•−^ to H_2_O_2_. Therefore, mitochondrial components may be potential therapeutic targets for overcoming the malignant effects of IR.
